# Conjunctivitis, Iritis, or Glaucoma?

**Published:** 1907-05-11

**Authors:** 


### Ophthalmology.

### CONJUNCTIVITIS, IRITIS, OR GLAUCOMA?

The hesitation which is often shown in the diagnosis of these common complaints, suggests that comparison of their symptoms may be helpful. The varieties of these diseases, which are liable to be confused, are acute catarrhal conjunctivitis, acute iritis, and acute glaucoma. The various forms of chronic conjunctivitis and chronic glaucoma seldom lead to any difficulty in differential diagnosis, though trachoma and chronic glaucoma are constantly overlooked, for want of eversion of the upper lid in the former case, and observation of the tension in the latter.

### A Comparison.

It will be well perhaps to take each main symptom or sign and compare it in the three diseases, finally classifying the signs in tabular form.

(a) *General or Constitutional Symptoms.*—Associated with conjunctivitis these are absent, except in patients who are liable to headaches from any ocular irritation, as from the presence of a foreign body in the conjunctival sac. In iritis there is generally fairly severe neuralgic pain radiating from the orbit, but there is no malaise or vomiting. In acute glaucoma, the constitutional symptoms in the active stage are very marked, the headache, vomiting, and raised temperature diverting the attention from the local signs, unless care be exercised.

(b) *Local Symptoms.*—In conjunctivitis there is a hot, gritty sensation beneath the lids and some pain, and photophobia may be very evident. In iritis the pain is a marked feature, often necessitating the application of leeches to the temple to relieve it. In acute glaucoma the pain is more severe still, but being somewhat masked by the general symptoms is apt to be relatively disregarded.

(c) *The lids* in conjunctivitis are congested and slightly swollen; in iritis generally normal; in glaucoma swollen a little.

(d) *Discharge.*—In conjunctivitis the discharge is purulent or muco-purulent, the patient relates that the lids are stuck in the morning on awaking from sleep. In iritis and glaucoma there is no discharge, but lachrymation is profuse.

(e) *The Conjunctiva* in simple acute inflammation is swollen and its transparency diminished. It is also injected, the engorgement of vessels being general, and those mainly affected being the larger ones. In iritis the conjunctiva is transparent and not swollen. Injection is seen, the small vessels especially being dilated. Moreover, careful inspection will detect a special " circum-corneal " zone of injection producing a violet tinge for two or three millimetres all round the corneal margin. On moving the conjunctiva by pressure of the finger through the lower lid, it will be seen that these vessels are not really conjunctival, but are situated in the episcleral tissue. This is an important sign in iritis, though it is present also in some inflammations of the cornea. In glaucoma there is injection and slight swelling.

(f) *The Cornea.*—In uncomplicated conjunctivitis the cornea is unaffected, and retains its transparency. In simple iritis the same is true, but when there is inflammation of the ciliary body also (iridocyclitis) there is a deposit on the posterior surface of the cornea—Descemet's membrane—of exudate arranged in dots of varying size, the part involved often assuming a triangular area with its base down, and its apex towards the centre of the cornea. In glaucoma a condition of œdema of the cornea occurs, so that the transparency of the tissue is interfered with, both the patient's vision and the view of the deeper tissues obtained by the surgeon, being impaired.

(g) *The Anterior Chamber.*—In conjunctivitis this is of normal depth, and the aqueous is clear. If the iritis be mild the same is again true, but if severe, exudate is poured out into the aqueous humour and produces cloudiness, and a layer of exudate falls to the bottom of the anterior chamber if the patient be in the erect attitude, producing a condition known as hypopyon. In glaucoma the aqueous is clear, but the depth of the anterior chamber is very much reduced, so that the iris seems to lie in direct apposition with the posterior surface of the cornea, especially at the periphery.

(h) *The Iris.*—In conjunctivitis this is normal, and active contraction is seen on exposing the eye to light. In iritis the pupil is small, the iris being swollen and discoloured. In glaucoma the iris is irregularly dilated and fixed; its colour is apparently not good, but this change is due to the defective transparency of the cornea.

(j) *Tension.*—The presence of increase of tension is a sign of great value, and a word or two may be said as to the method of estimating it. The patient is instructed to turn the eyes down, slightly but not excessively, as this latter would be likely to cause a rise of tension from pressure of the ocular muscles on the globe. The surgeon's hands are then steadied by placing the fifth and fourth finger of each hand on some part of the head around the orbit so that the second fingers can reach the upper lid comfortably. With these, or, if preferred, with the first finger of each hand, the tension is estimated through the upper lid, somewhat as fluctuation is sought for in any other situation, though with this difference, that the eyeball is indented with each finger alternately instead of, as in testing for fluctuation, one finger being kept still as the index. If only one eye is affected the tension of the sound eye is first noted to form a ground of comparison, but practice soon gives the delicacy required to give an opinion even on one eye as to whether its tension is normal, increased, or diminished. In conjunctivitis tension is normal. In acute iritis the same is true, but if the iris is allowed to become totally adherent to the lens, the tension rises subsequently. In acute glaucoma the tension is so much raised that there is no sense of fluctuation at all, and the eye feels as hard as a stone.

(k) *Vision.*—In conjunctivitis vision is only impaired, if at all, by the mucus over the cornea. In iritis vision will be fair until exudate in the anterior chamber interferes with it. In glaucoma, if the attack has lasted a day or two, vision is less than one-tenth of the normal, and rapidly decreases to mere perception of light.

These signs may be tabulated thus:—

| | Conjunctivitis. | Iritis. | Glaucoma. |
|---|---|---|---|
| *a.* General symptoms | None | Very slight | Severe |
| *b.* Local ... | Slight gritty feeling | More pain—radiating | Intense pain |
| *c.* Lids ... | Congested, swollen | Normal | Some swelling |
| *d.* Discharge... | Pus, or muco-pus | Tears | Tears |
| *e.* Conjunctiva | Injected, swollen, dull | Injection especially circum-corneal, not swollen or dull | Injection and some swelling |
| *f.* Cornea ... | Normal | Normal, unless severe or complicated | "Steamy" |
| *g.* Anterior chamber | Normal | Normal, or with exudate | Very shallow or absent |
| *h.* Iris... ... | Normal | Contracted, fixed, discoloured, swollen | Irregularly dilated, slightly discoloured |
| *j.* Tension ... | Normal | Normal | Stony hard |
| *k.* Vision ... | Good | More likely to be impaired | Very bad |

